# Nucleoside deaminases: the key players in base editing toolkit

**DOI:** 10.52601/bpr.2023.230029

**Published:** 2023-12-31

**Authors:** Jiangchao Xiang, Wenchao Xu, Jing Wu, Yaxin Luo, Bei Yang, Jia Chen

**Affiliations:** 1 Gene Editing Center, School of Life Science and Technology, ShanghaiTech University, Shanghai 201210, China; 2 Shanghai Institute for Advanced Immunochemical Studies, ShanghaiTech University, Shanghai 201210, China; 3 Shanghai Clinical Research and Trial Center, Shanghai 201210, China

**Keywords:** Nucleoside deaminase, Base editors

## Abstract

The development of nucleoside deaminase-containing base editors realized targeted single base change with high efficiency and precision. Such nucleoside deaminases include adenosine and cytidine deaminases, which can catalyze adenosine-to-inosine (A-to-I) and cytidine-to-uridine (C-to-U) conversion respectively. These nucleoside deaminases are under the spotlight because of their vast application potential in gene editing. Recent advances in the engineering of current nucleoside deaminases and the discovery of new nucleoside deaminases greatly broaden the application scope and improve the editing specificity of base editors. In this review, we cover current knowledge about the deaminases used in base editors, including their key structural features, working mechanisms, optimization, and evolution.

## INTRODUCTION

Eukaryotic genomes are composed of billions of major nucleobases. While the modifications of these bases can lead to functional diversity, those unexpected base mutations could cause genomic instability (Korf *et al.* 2019). Therefore, developing precise and efficient tools to achieve base conversion in DNA or RNA molecules has been a long-sought goal (Doudna 2020). Since its advent, the clustered regularly interspaced short palindromic repeat (CRISPR)-associated protein (Cas) systems have been widely applied in gene editing. The canonical CRISPR-Cas9 system contains a Cas9 protein whose DNA-targeting specificity and cutting activity are programmed by a short guide RNA (Doudna and Charpentier 2014; Jinek *et al.* 2012; Mali *et al.* 2013), establishing a platform for more versatile gene editing. Later on, base editors (BEs), a more advanced gene editing toolkit, were developed to achieve precise and efficient editing at the single-base level, without triggering double-strand breaks (DSBs) or requiring donor DNA templates (Gaudelli *et al.* 2017; Gehrke *et al.* 2018; Komor *et al.* 2016; Nishida *et al.* 2016; Wang *et al.* 2017, 2018, 2020, 2021). Genetic manipulation at the single-base level enables scientists to study gene function or correct disease-causing mutations, which holds tremendous value not only for basic research but also for disease treatment (Wang and Doudna 2023).

**Figure 1:**
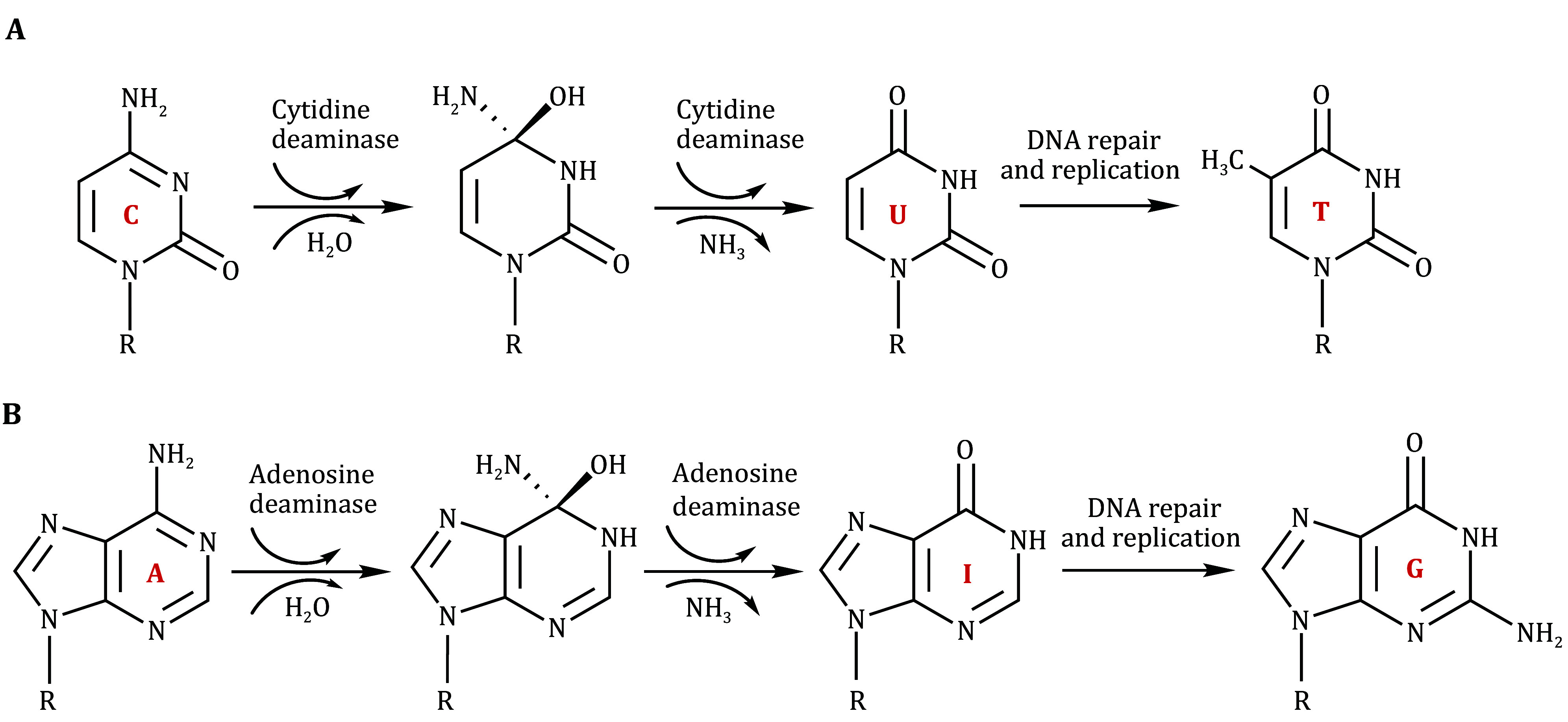
Cytidine and adenosine deamination processes. **A** Cytidine deamination generates uridine, which is read as thymidine by DNA polymerase. C, cytidine; U, uridine; T, thymidine. R represents 2´-deoxyribose in DNA or ribose in RNA. **B** Adenosine deamination generates inosine, which is read as guanosine by DNA polymerase. A, adenosine; I, inosine; G, guanosine. R represents 2´-deoxyribose in DNA or ribose in RNA

**Figure 2:**
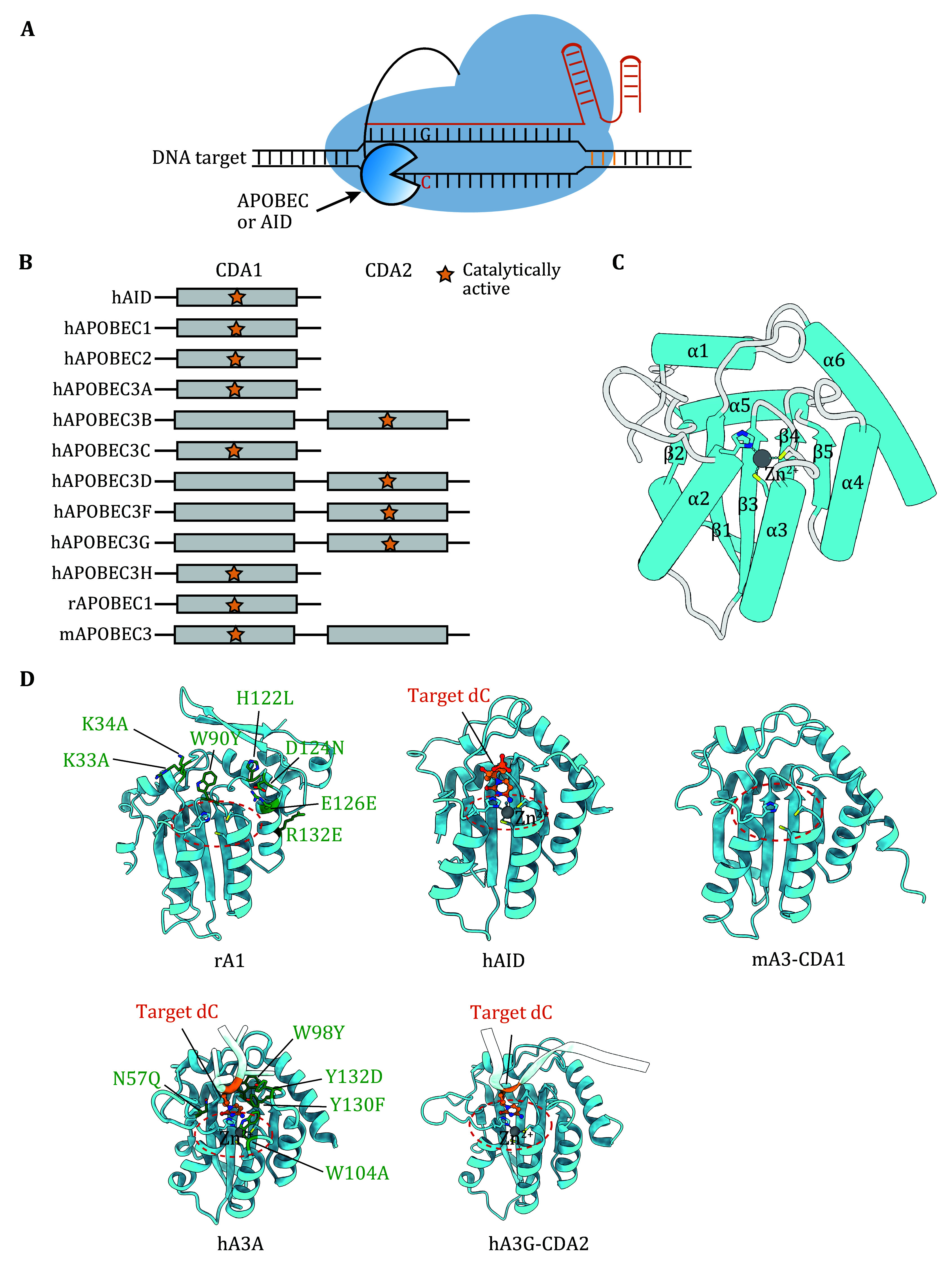
The conserved core cytidine deaminase domain of AID/APOBEC family. **A** Schematic illustration of AID/APOBEC-derived CBE. **B** Schematic of the AID/APOBEC family. Each member of the family contains the core catalytically active zinc-dependent cytidine deaminase domain (CDA), star labeled. **C** Cartoon topology of hA3A (PDBID: 5KEG) illustrating the typical core CDA fold shared by the AID/APOBECs family. The CDA fold is composed of a five-strand β-sheet (β1–β5) surrounded by six α-helices (α1–α6). **D** Cartoon representations of rA1 (Uniprot: P38483, generated from Alphafold2), hAID (PDBID: 5W0U), mA3-CDA1 (Uniprot: Q99J72, generated from Alphafold2), hA3A (PDBID: 5KEG), and hA3G-CDA2 (PDBID: 6BUX) structures. Target dC located at the bottom of catalytic pocket was showed as ball and stick models and colored in orange. Zn ion is depicted as a grey sphere. Positions of the engineered residues in optimized CBEs were highlighted with green sticks. Red dash circles indicate the catalytically active pocket

BEs contain two primary components: a programmable DNA-binding protein (locator), such as a catalytically impaired Cas nuclease, and a DNA-modifying enzyme (effector), such as nucleoside deaminases (Yang and Chen 2020). BEs can be classified as cytosine base editors (CBE) and adenine base editors (ABE) according to the nucleoside deaminases they contain (Huang *et al.* 2021a). Uridine (U) and thymidine (T) can be formed by the spontaneous hydrolytic deamination of cytidine (C) and 5-methylcytidine, respectively. In humans, C-to-U deamination can also be catalyzed by numerous cytidine deaminases, the best known of which belong to a family of mammalian enzymes called “activation-induced cytidine deaminase/apolipoprotein B mRNA-editing enzyme catalytic polypeptide-like (AID/APOBEC) protein family” (Wedekind *et al.* 2003; Yang *et al.* 2017) (Figs. 1A and 2B). Adenosine (A), like cytidine, contains an exocyclic amino group, and deamination changes its pairing properties. Deamination of adenosine to inosine (A-to-I) in RNA can be catalyzed by the adenosine deaminase acting on RNA (ADAR) protein family (Savva *et al.* 2012) (Fig. 1B). In addition, other nucleoside deaminases from prokaryotic organisms were also discovered, *e.g.*, transfer RNA (tRNA) adenosine deaminase (TadA) (Kim *et al.* 2006; Wolf *et al.* 2002) and double-strand DNA-specific deaminase toxin A (DddA) (Mok *et al.* 2020), the members of which have been utilized to develop gene-editing tools as well. Notably, DddA targets double-stranded DNA (dsDNA) instead of single-stranded DNA (ssDNA) (Mok *et al.* 2020; Salter and Smith 2018), enabling it to fulfill editing goals at places where other deaminases cannot reach (Kim and Chen 2023). More recently, AI-based protein structure prediction and clustering established a suite of ssDNA deaminases and dsDNA deaminases, further enriching the deaminases tool family (Huang *et al.* 2023).

Thus, nucleoside deaminase, the effector with base modifying activity, plays determinant roles in the efficiency, scope, accuracy, and specificity of base editing (Yang and Chen 2020). Extensive studies on the discovery, engineering, and evolution of nucleoside deaminases have significantly enlarged the BE toolkit (Barrera-Paez and Moraes 2022; Huang *et al.* 2021a; Yang *et al.* 2019). In this review, we summarize the cytidine and adenosine deaminases that have been widely applied in the base editing field and highlight the structural and functional features of the native enzyme and their engineered variants, which have led to the development of more efficient and precise BEs.

## CYTIDINE DEAMINASE

### APOBEC/AID, ssDNA deaminases

Each member of the APOBEC family has specific physiological functions that involve the binding of nucleic acid and catalysis of cytidine to uridine deamination in the context of either RNA and/or ssDNA (Salter *et al.* 2016). In human cells, the APOBEC family consists of 11 genes, *i.e.*, APOBEC1 (A1), AID, APOBEC2 (A2), APOBEC3A–H (A3A–H), and APOBEC4 (A4). These genes and their alternatively spliced variants can produce various protein products. These deaminases all contain at least one catalytic domain that comprises canonical zinc-dependent deaminase signature motif (H/C-X-E-X_23-28_-P-C-X_2-4_-C (HECC) (Pecori *et al.* 2022) (Fig. 2B). A number of structures of APOBECs have been reported without nucleic acid or nucleotide ligands bound, including that of the single cytidine deaminase domain (CDA) domain-containing APOBECs like hA1, hAID, hA3A, hA3C, and hA3H, as well as that of the C terminal catalytic domain of the dual CDA domain-containing APOBECs like hA3B, hA3F and hA3G (Salter and Smith 2018). These structures all adopt a typical CDA fold wherein a five-strand β-sheet (β1–β5) is surrounded by six α-helices (α1–α6). Of the loops (L1 to L10) that connect the β-stands and α-helices, loops 1, 3, 5 and 7 participate in the formation of the substrate binding groove wherein the catalytic zinc ion is coordinated by the His-Glu (H and E) and Cys-Cys (C) motifs on α2 and L5/α3, respectively (Fig. 2C, Zn gray sphere). Most A3 enzymes prefer a 5’-TC (or UC) dinucleotide sequence in ssDNA or single-stranded RNA substrates, except for A3G which prefers to deaminate cytidines in a 5’-CC motif (Table 1) (Pecori *et al.* 2022; Salter *et al.* 2016).

**Table 1:** Types and characteristics of cytidine deaminases in CBEs

Cytidine deaminases	CBEnames	Engineering sites	Features	Target C preference	Reference
rAPOBEC1	BE3	WT	Canonical CBE architecture	5’-TC	Komor *et al.* 2016
	YE1-BE3 EE-BE3 YE2-BE3 YEE-BE3	W90Y, R126E R126E R132E W90Y, R132E W90Y, R126E, R132E	Showed constricted editing windows and reduced bystander editing compared to BE3	5’-TC	Kim *et al.* 2017
	SECURE	R33A, K34A	Reduced off-target RNA editing compared with BE3	5’-TC	Grunewald *et al.* 2019a
hAID	CRISPR-X	WT	Broad mutagenesis, increase in C-to-non-T edits relative to BE3	None	Hess *et al.* 2016,
	TAM	WT	Broad mutagenesis and C-to-non-T edits relative to BE3	None	Ma *et al.* 2016
PmCDA1	Target-AID	WT	Exhibits altered editing windows relative to N-terminal deaminase fusion relative to BE3	None	Nishida *et al.* 2016
hAPOBEC3A	eA3A-BE3	N57G	Reduced bystander editing and reduced off-target RNA-editing activity relative to BE3	5’-TC	Gehrke *et al.* 2018
	hA3A-BE3-Y130F hA3A-BE3-Y132D	Y130F Y132D	Narrower editing windows and reduced bystander editing	5’-TC	Wang *et al.* 2018
	hA3A-BE3-Y130F/Y132D	Y130F, Y132D	Narrowed editing windows		
	BEACON1 BEACON2	W98Y, Y132D W98Y, W104A, Y130F	Narrowed editing window and induce low levels of indels	5’-TC	Wang *et al.* 2020
hAPOBEC3G	hA3G-BE	C-terminal catalytic domain of hA3G	Preferentially at 5’-CC-3’ motifs	5’-CC	Liu *et al.* 2020
mAPOBEC3	tBE	N-terminal catalytic domain of mA3	Eliminated OT mutations	5’-TC	Wang *et al.* 2021
Sdd3	/	WT	Expanded sequence context preference and lower off-target activities	5’-GC or 5’-AC	Huang *et al.* 2023
Sdd6	/	WT	Showed no strong sequence context preference and nearly no off-target editing activity.	None	
Sdd7	/	WT	Showed no strong sequence context preference.	None	

In 2016, Komor *et al*. developed the first series of base editors by fusing rat APOBEC1 (rA1) to dCas9 (catalytical dead Cas9) or nCas9 (Cas9 nickase), thereby leveraging these cytidine deaminases for programmable and precise base editing (Fig. 2A). Their final optimized construct was named BE3, which achieved C-to-T single base editing with low levels of unwanted indels, as it avoids generating DSBs (Komor *et al.* 2016). Later, BEs like YE1-BE3(W90Y + R126E), YE2-BE3(W90Y + R132E), EE-BE3(R126E + R132E) and YEE-BE3(W90Y + R126E + R132E) were developed by mutating the key residues in rA1 that interact with DNA (Fig. 2D). These mutations can narrow BE’s editing window from 5 nt to 1–2 nt, albeit with slightly reduced editing efficiency at target C (Table 1) (Kim *et al.* 2017). Grünewald *et al*. introduce two neutralizing amino acid mutations, R33A and K34A, into active-site loop 1 of rA1, which reduced off-target RNA editing compared with BE3 (Fig. 2D).

Since the introduction of the BE3 system, more BEs have been developed through optimizing or replacing the deaminase moiety. For instance, several cytidine deaminases other than rA1 including hAID (Hess *et al.* 2016; Ma *et al.* 2016), PmCDA1 (Nishida *et al.* 2016), hA3A (Gehrke *et al.* 2018; Wang *et al.* 2018), hA3B (Doman *et al.* 2020), hA3G-CDA2 (Liu *et al.* 2020) and mouse APOBEC3 (mA3) (Wang *et al.* 2021) have been put into the conventional BE3 architecture. As CpG methylation generally has a negative effect on the C-to-T editing efficiency by rA1-based BE3, Wang *et al*. replaced the rA1 moiety with hA3A and demonstrated that the hA3A-BE3 is the most efficient at methylated CpG sites among BEs that follow the conventional BE3 architecture (Wang *et al.* 2018). To narrow the editing window of hA3A-BE3, Wang *et al.* further introduced the Y130F or Y132D mutation into the deaminase, both of which are in loop 7 and predicted to interact directly with the nucleic acid substrate (Fig. 2D) (Wang *et al.* 2018). Besides, through AI-assisted structure prediction, Huang *et al*. identified some novel deaminases with disparate deamination motif preferences on ssDNA substrates, including Sdd3, Sdd6, and Sdd7, which would further expand the editing scope of base editors (Table 1). Though these Sdds share a core structure similar to DddA and theoretically do not belong to the APOBEC/AID family, they showed more robust cytosine base editing activity on ssDNA than some APOBEC/AID deaminases and could be used to develop base editing tools (Huang *et al.* 2023).

Meanwhile, BEs with novel architecture were also developed, of which the transformer base editor system (tBE) solved the off-target problem of conventional BEs through its ingenious design. Finding that the inactive CDA domain of mA3 functions as a deoxycytidine deaminase inhibitor (dCDI), Wang *et al.* took advantage of mA3dCDI to develop the tBE system. tBE remains inactive at off-target sites due to the fusion of a cleavable mA3dCDI, but would be transformed into deamination-competent form by cleaving off dCDI at on-target sites, therefore eliminating OT mutations (Wang *et al.* 2021).

All in all, the APOBEC/AID family members and other ssDNA deaminases have been exploited to play critical roles in various base editors.

### DddA

Compared to APOBEC/AID family, DddA has a unique ability to catalyze the direct deamination of cytidine in dsDNA. Its distinct dsDNA binding and deamination activities have also brought new opportunities for gene editing. Previously, the lack of feasible nucleic acid delivery systems across the mitochondrial double-membranes hindered the application of CRISPR-based DNA editing tools within mitochondria. Therefore, the development of novel mitochondrial base editing tools to repair mutant mitochondrial genomes and manipulate mitochondrial gene expression becomes particularly challenging.

**Figure 3:**
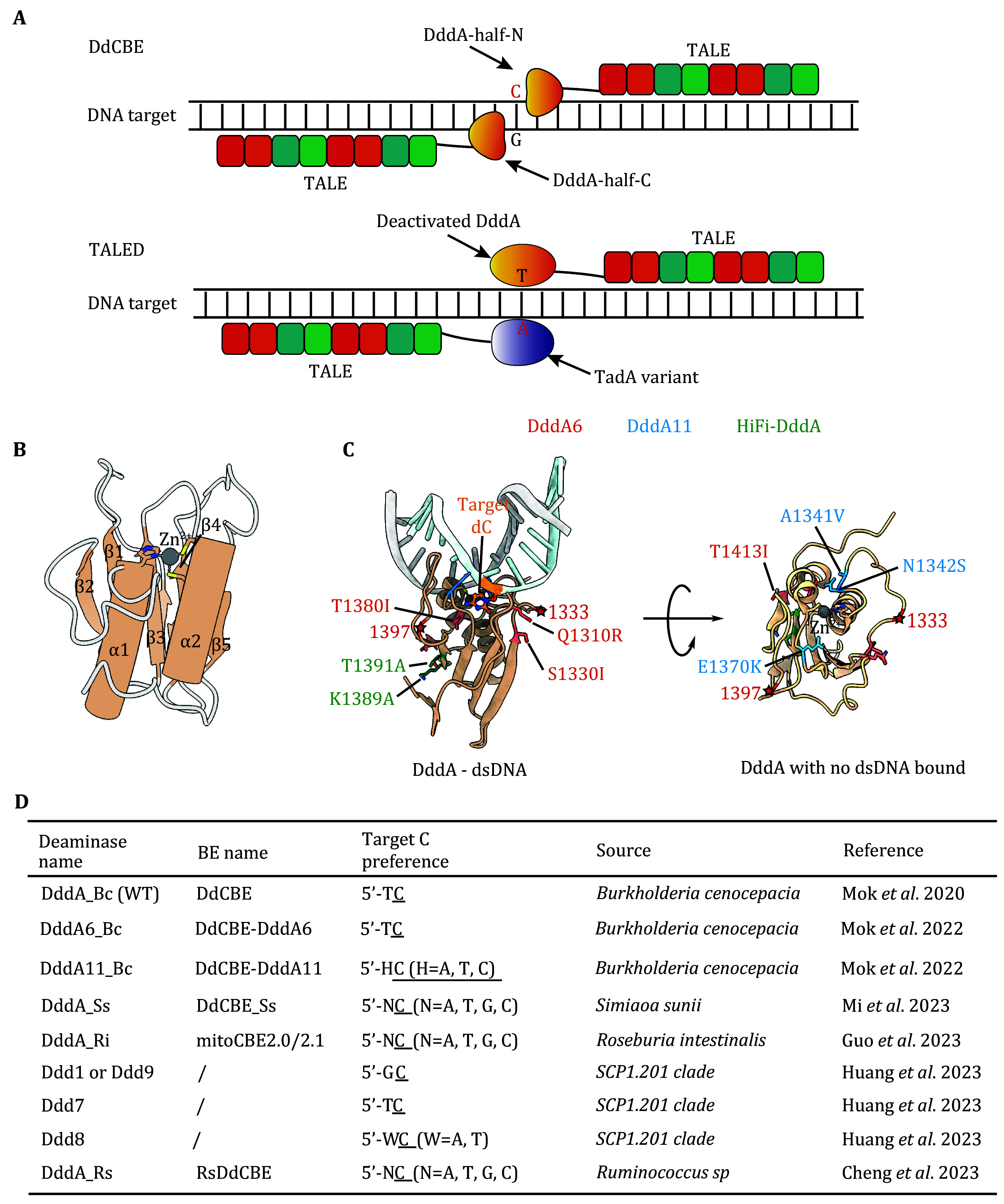
Deaminase toxin A (DddA) and its characteristics in genomic DNA and mitochondrial DNA editing. **A** Schematic illustration of the design of DddA-derived cytosine base editor (DdCBE) and TALE-linked Deaminase (TALED). **B** Cartoon topology of a DddA (PDBID: 8E5E) shows that the conserved core CDA fold is composed of a five-strand β-sheet (β1–β5) and two α-helices (α1 and α2). **C** Cartoon representations of DddA in complexed dsDNA substrate (PDBID: 8E5E). Target d**C** located at the bottom of the catalytic pocket is shown as ball and stick models and colored in orange. Zn ion is depicted as a grey sphere. Positions of engineered residues in DddA6, DddA11 and HiFi-DddA are highlighted with sticks and colored in salmon, blue, and green respectively. The side view (left) with dsDNA and the top view without dsDNA (right) were both shown. **D** Substrate preferences of DddA, its engineered variants and newly discovered homologs, which were all used to develop more advanced mitochondrial BEs

In 2020, a novel bacterial toxin, DddA, was identified in *Burkholderia cenocepacia*. Its C-terminal toxin domain (DddA_tox_) acts as a dsDNA deaminase, deaminating cytosine in dsDNA to form uracil (Mok *et al.* 2020). To overcome the cytotoxicity of the full-length toxin-like protein, DddA_tox_ is further divided into two parts, DddA_tox_-N and DddA_tox_-C. Both parts are then fused with a mitochondrial targeting signal sequence (MTS) and a TALE (transcription activator-like effector) array to generate DdCBE. One TALE array (TALE-L) can bind to the left side of the target site and the other TALE array (TALE-R) can bind to the right side of the target side, thereby reconstituting a functional DddA at the target site (Fig. 3A). Leveraging the targeting activity of TALE and the dsDNA deamination activity of DddA, efficient C-to-T editing of mtDNA target sites has been achieved by DdCBE (Mok *et al.* 2020). Furthermore, by fusing another effector, *i.e.*, adenosine deaminase with DddA, a new mitochondrial genome editor TALED was developed to induce A-to-G editing (Fig. 3A) (Cho *et al.* 2022).

Although DddA differs from the APOBEC/AID family in terms of protein sequence and native function, the DddA structure surprisingly also adopts a typical deaminase fold, including five-strand β-sheet (β1–β5), two α-helices (α1–α2) and active site histidine and cysteine residues in position to coordinate a zinc ion (Fig. 3B). The naturally preferred substrates of DddA are 5’-TC dinucleotides, similar to that of most APOBECs (Mok *et al.* 2020; Pecori *et al.* 2022). Through bacteriophage-assisted continuous evolution (PACE) and bacteriophage-assisted non-continuous evolution (PANCE), Mok *et al*. also obtained improved DddA variants, DddA6 (Q1310R, S1330I, T1380I and T1413I) and DddA11 (S1330I, A1341V, N1342S, E1370K, T1380I, and T1413I) (Mok *et al.* 2022). DddA6 manifests enhanced editing at 5’-TC motif and DddA11 exhibits expanded editing scope to 5’-HC (H=T/A/C), respectively (Fig. 3C). Specifically, the alteration of A1341V, N1342S, and E1370K in DddA11 reshapes the pocket contacting the –1 T base, which may be critical for expanding the targeting scope of DddA11 (Mok *et al.* 2022).

To reduce off-target editing of DdCBE, Lee *et al*. introduced T1391A and K1389A in the binding interface of DddA_tox_-N and DddA_tox_-C and constructed HiFi-DdCBE (Fig. 3C). HiFi-DdCBE largely maintains the activity of DdCBE while improving its specificity, thus achieving high efficiency and precision suitable for therapeutic applications (Lee *et al.* 2023). In addition, new DddA homologs have also been discovered and reported by different groups, providing diverse choices for constructing mitochondrial base editors. For example, a new DddA homolog named DddA_Ss was identified from *Simiaoa sunii*, which has been used to develop new DdCBE_Ss to enable editing at the 5’-GC context in dsDNA (Mi *et al.* 2023). Guo *et al*. also identified a novel DddA homolog from *Roseburia intestinalis* and referred to it as DddA_Ri. They successfully developed CRISPR-based nuclear genome cytosine base editors (crDdCBE) and TALE-based mitochondrial genome cytosine base editors (mitoCBE) with DddA_Ri, achieving efficient dsDNA editing in nuclear and mitochondrial genomes respectively. Compared to DddA11, DddA_Ri-derived mitoCBE completely overcomes the 5’-TC context limitation (Guo *et al.* 2023). Recently, Huang *et al*. used AlphaFold2’s structural classification feature to identify many new DddA-like clades, which have substrate preference distinct from 5’-TC. Among them, the newly identified Ddd1 and Ddd9 exhibit higher activity at the 5’-GC motif (Huang *et al.* 2023). These newly discovered DddA proteins greatly enriched the mitochondrial base editing toolkits (Cheng *et al.* 2023; Kim and Chen 2023) (Fig. 3D). These studies also highlight the existence of dsDNA deaminases with diverse editing characteristics, which deserve to be further explored and applied.

## ADENOSINE DEAMINASE

### TadA-WT and TadA variant

**Figure 4:**
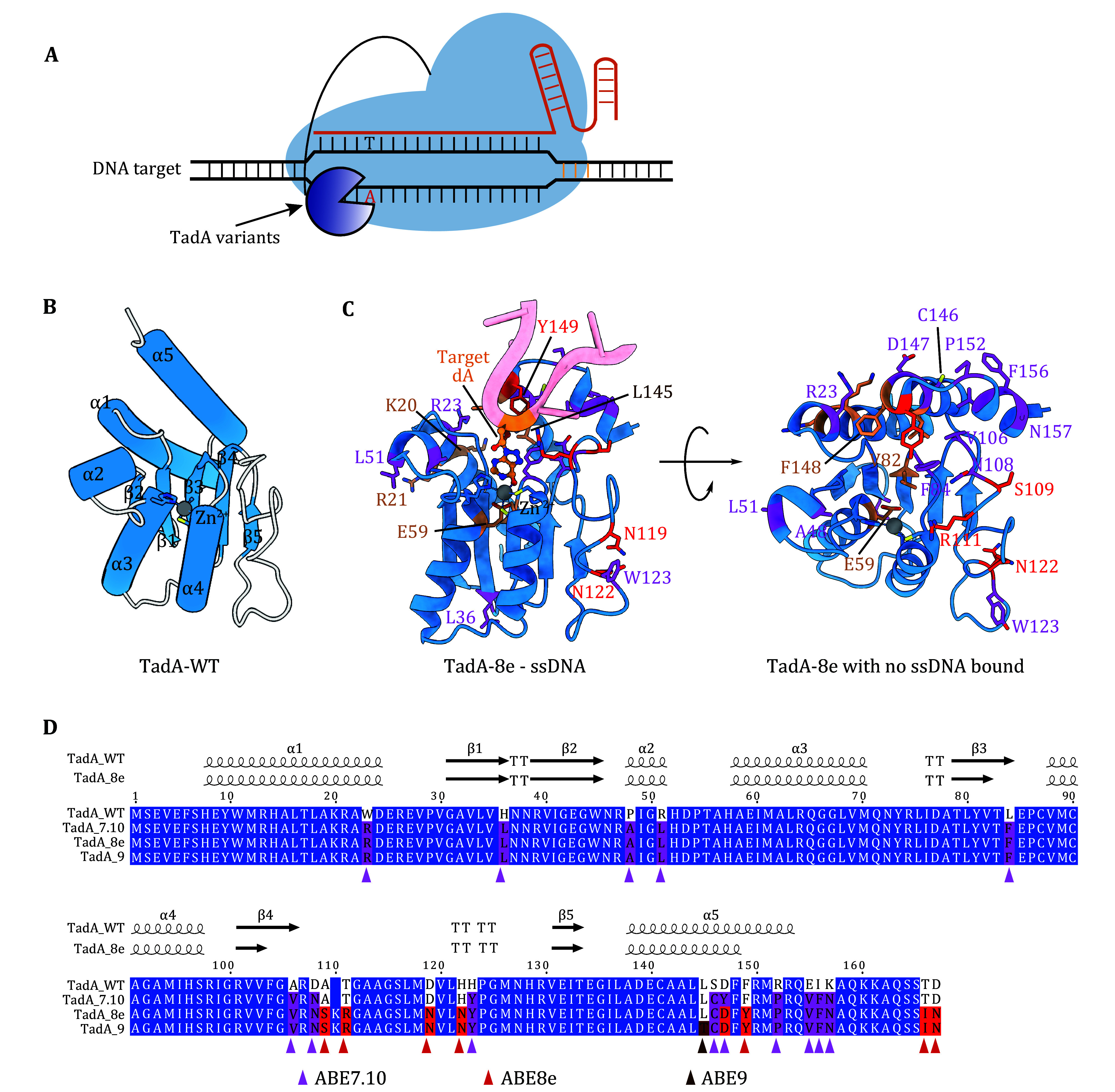
Structural analysis of TadA and TadA variants in ABE. **A** Schematic illustrating the design of TadA-derived adenine base editor (ABE). Evolved TadA variants can deaminate adenosines in ssDNA to yield inosines, which are read as guanosines by DNA polymerase. **B** Cartoon topology of a TadA-WT (PDBID: 1Z3A) shows that the core CDA fold is composed of a five-strand β-sheet (β1–β5) and five α-helices (α1–α5). **C** Cartoon representations of TadA-8e in complex with NTS DNA (partial sequence shown). The evolved residues are shown as sticks and colored purple (TadA-7.10) or red (TadA-8e) (PDBID: 6VPC). The side view (left) with ssDNA and the top view without ssDNA substrate (right) are both shown. **D** Sequence alignment of the TadA and TadA variants (TadA-7.10, TadA-8e and TadA-9). The secondary structure elements (α-helices and β-strands) of the TadA-WT (PDBID:1Z3A) and TadA-8e (PDBID:6VPC) are shown above the alignment. The mutations introduced during the directed evolution of ABE7.10, ABE8e and ABE9 are labeled in purple, red, and brown triangles respectively

Inspired by CBE, another type of genome editing technology capable of altering A to G was developed. However, native adenosine deaminase can only deaminate free adenosine, the adenosine in single-stranded RNA or the adenosine in the RNA of mismatched RNA-DNA heteroduplexes, but has no activity on adenosine in dsDNA or ssDNA (Zheng *et al.* 2017). To establish an ABE, *Escherichia coli* TadA was selected for seven rounds of directed evolution *in vitro* to obtain TadA* (hereafter referred to as TadA-7.10) that exhibits adenosine deamination activity in ssDNA. The authors then artificially constructed a TadA-WT: TadA-7.10 heterodimeric adenosine deaminase, which, when fused with nCas9 (D10A), generated ABE7.10. ABE7.10 exhibited efficient A-to-G editing within its editing window (A4–A7) in mammalian cells (Gaudelli *et al.* 2017) (Fig. 4A). Afterward, ABE7.10 was further evolved to generate ABE8e, which carries a single TadA domain (TadA-8e) and deaminates DNA at a higher rate than ABE7.10 (Richter *et al.* 2020). Notably, the TadA-8e moiety carries 8 additional mutations as compared to TadA-7.10 (Fig. 4C and 4D, Table 2).

**Table 2:** Types and characteristics of TadA variants in ABEs

Deaminase name	BE name	Engineering sties	Features	Reference
TadA-7.10	ABE7.10	W23R; H36L, P48A, R51L, L84F, A106V, D108N, H123Y, S146C, D147Y, R152P, E155V, I156F, K157N	Exhibited efficient A-to-G editing within its editing window (A4–A7) in mammalian cells	Gaudelli *et al.* 2017
TadA-7.10- F148A, V106W	ABE7.10- F148A, V106W	TadA-7.10 + F148A, V106W	Reduce the RNA off-targeting of ABEs	Zhou *et al.* 2019
TadA-minABEmax	miniABEmax	TadA-7.10 + K20A/R21A, V82G	Reduce the RNA off-targeting of ABEs	Grunewald *et al.* 2019b
TadA-8e	ABE8e	TadA-7.10 + A109S, T111R, D119N, H122N, S146C, F149Y, T166I, D167N	Deaminate DNA at higher rate than ABE7.10	Lapinaite *et al.* 2020, Richter *et al.* 2020
TadA-9	ABE9	TadA-8e + L145T	ABE9 has a narrower editing window compare to ABE7.10 and ABE8e	Chen *et al.* 2023

The structure of TadA-WT also exhibits the basic characteristics of deaminase enzymes, including a five-strand β-sheet (β1–β5), flanked by two α-helices (α1 and α5) on one side and three α-helices (α2–α4) on the opposite side. Likewise, key residues from the α3–α4 helices and surrounding loops coordinate the essential zinc ion and form the catalytically active site (Fig. 4B). The recent cryo-EM structure of the DNA-bound ABE8e machine presents how TadA-8e captures the ssDNA while excluding more rigid tRNA substrates (Lapinaite *et al.* 2020; Losey *et al.* 2006). Relative to TadA-WT, TadA-7.10 and TadA-8e contain 14 and 22 substitutions, respectively (Fig. 4D). The 14 mutations in TadA-7.10 predominantly locate in the substrate-binding loops (W23R; P48A, R51L, L84F, A106V, D108N, and H123Y) and the C-terminal α-5 helix (S146C, D147Y, R152P, E155V, I156F, and K157N) (Fig. 4C). Among them, the D108N mutation is critical by increasing the binding affinity between ssDNA and TadA-7.10, as reversion of this single mutation abolishes the adenosine activity of TadA-7.10 on ssDNA. Notably, the R152P mutation breaks the α-5 helix in both TadA-7.10 and TadA-8e, making them more accessible to ssDNA (Lapinaite *et al.* 2020). Compared to TadA-7.10, two mutations (T111R and F149Y) in TadA-8e stabilize a U-shape flipped conformation of the nontarget strand (NTS) DNA substrate and further facilitate its fitting into the deaminase active center (Fig. 4C) (Lapinaite *et al.* 2020).

The TadA-WT enzyme physiologically deaminates adenine 34 (A34) in *E. coli* transfer RNA (tRNA)^*Arg2*^, and the TadA variants used in ABEs had not completely lost their RNA editing activity (Kim *et al.* 2006; Rees *et al.* 2019). Therefore, to reduce the RNA off-targeting of ABEs, some critical mutations were introduced to TadA, including K20A/R21A and V82G in two versions of miniABEmax (Grunewald *et al.* 2019b) (Table 2), F148A mutation (Zhou *et al.* 2019) and V106W mutation (Rees *et al.* 2019) in the deaminase domain of TadA-7.10. The residues K20 and R21 are solvent exposed in the α1-helix and their substitution to alanine changes the positive charge of this surface. This probably reduces the interaction between the deaminase domain and RNA, and thus, reduces RNA deamination in cells. Since V82 and V106 both located at the bottom of the catalytic pocket, their substitution likely reshapes the active center, resulting in decreased activity on RNA (Fig. 4C) (Lapinaite *et al.* 2020).

Chen *et al*. employed a structure-based mutagenesis strategy and identified the L145T mutation to develop ABE9. In comparison to ABE8e, ABE9 has a narrower editing window (Chen *et al.* 2023). The mutations in ABE8e and ABE9 are primarily located in the active site loops and the C-terminal α-helix, similar to the mutations in ABE7.10 (Fig. 4D, Table 2). Unlike the plethora of ssDNA-editing cytidine deaminases in the APOBEC/AID family and other clades, the lack of adenine deaminase that naturally acts on ssDNA will bring more challenges to the further development of ABE.

### ADAR family

Two different enzymes carry out A-to-I editing in humans: ADAR1 and ADAR2 (Bass *et al.* 1997). ADARs share a common domain architecture, consisting of a variable number of amino-terminal dsRNA binding domains (dsRBDs) and a carboxy-terminal catalytic deaminase domain (Goodman *et al.* 2012). Similar to TadA, ADAR targets adenosines in double-stranded RNA (dsRNA) and deaminates them into inosines, which are biochemically interpreted as guanosines, thereby introducing functional A-to-G mutations into RNA (Bass and Weintraub 1988; Savva *et al.* 2012).

**Figure 5:**
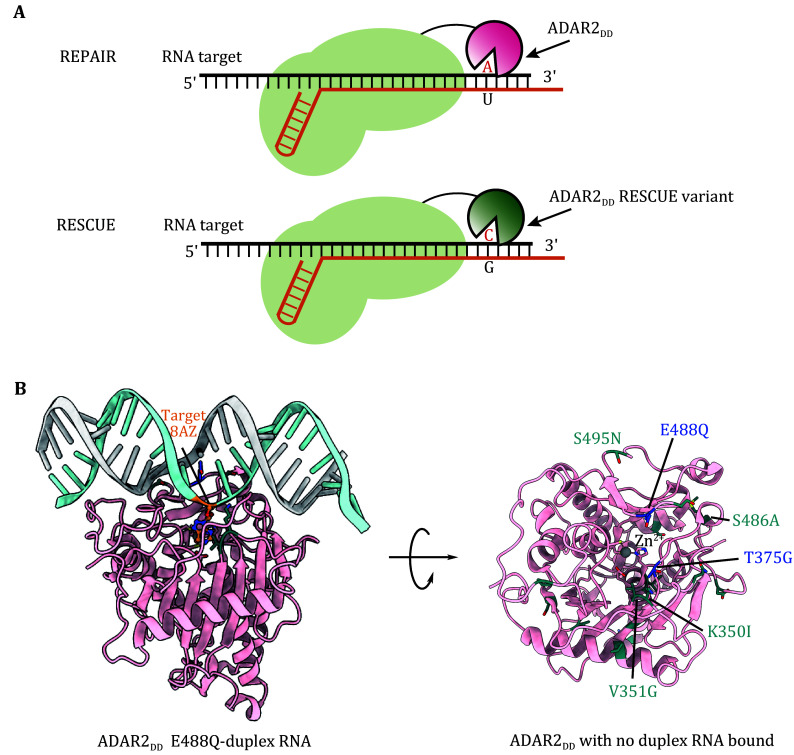
Adenine base editing in RNA. **A** Schematic of RNA editing by dCas13b-ADAR2_DD_ fusion proteins (REPAIR) or dCas13b-ADAR2_DD_ variants fusion proteins (RESCUE). **B** Structure of ADAR2_DD_ E488Q bound to the duplex RNA (PDBID:5ED1). Positions of evolved key residues in the RESCUE system are shown as green sticks. The side view with ssDNA (left) and the top view without ssDNA substrate (right) are both shown

Cox *et al*. fused the adenine deaminase domain of ADAR2 (ADAR2_DD_) with the catalytically inactive Cas13 protein, which enabled A-to-I RNA editing at the transcriptome level, and named the editor as RNA Editing for Programmable A to I Replacement (REPAIR) (Cox *et al.* 2017) (Fig. 5A). Furthermore, in order to minimize the substantial off-target RNA editing associated with the first version of REPAIR (REPAIRv1), they introduced E488Q/T375G into ADAR2_DD_ and developed REPAIRv2, which can induce specific and efficient A-to-I base editing in RNA (Cox *et al.* 2017) (Fig. 5B, Table 3).

**Table 3:** Types and characteristics of ADAR deaminases used in RNA editing

Deaminase name	BE name	Engineering sties	Features	Reference
ADAR2_ DD_	REPAIR	E488Q	Enable A-to-I RNA editing at the transcriptome level	Cox *et al.* 2017
ADAR2_DD_	REPAIRv2	E488Q, T375G	Induce specific and efficient A-to-I base editing in RNA	Cox *et al.* 2017
ADAR2_DD_	RESCUEr3	E488Q, V351G, S486A, T375S	Improve C-to-U deamination activity	Abudayyeh *et al.* 2019
ADAR2_DD_	RESCUE	E488Q, V351G, S486A, T375S, S370C, P462A, N597I, L332I, I398V, K350I, M383L, D619G, S582T, V440I, S495N, K418E, S661T	Significantly increased C-to-U deamination activity at all tested targets in the context of any flanking 5' and 3' bases while retaining A-to-I editing activity.	Abudayyeh *et al.* 2019

For C-to-U RNA base editing, Abudayyeh *et al*. focused on the ADAR2_DD_ residues contacting RNA substrates and performed three rounds of rational mutations on ADAR2_DD_ fused with a catalytically inactive Cas13b homolog (Abudayyeh *et al.* 2019) (Fig. 5B). This effort resulted in RESCUEr3 (RESCUE round 3), which exhibited improved C-to-U editing activity. Building upon this, they initiated directed evolution within ADAR2_DD_ to identify additional candidate mutations that would enhance RESCUE activity in yeast. After 16 rounds of evolution, they ultimately obtained the final construct, RESCUEr16 (hereafter referred to as RESCUE), which manifested significantly increased C-to-U deamination activity at all tested targets in the context of any flanking 5' and 3' bases while retaining A-to-I editing activity (Abudayyeh *et al.* 2019). The major mutations of RESCUE are shown in the structure of ADAR2_DD_ with no duplex RNA bound as a green stick model (Fig. 5B). Mutations introduced to the catalytic core (V351G and K350I) and to the regions contacting the RNA target (S486A, S495N) are both essential to RESCUE activity (Table 3). Huang *et al*. fused human A3A (hA3A) with dPspCas13b to create a distinct C-to-U RNA editing tool called C-to-U RNA Editor (CURE), which incorporates an editing-enhancing mutation Y132D (Huang *et al.* 2021b). Unlike RESCUE, CURE is designed exclusively for C-to-U editing and does not perform A-to-I editing. Furthermore, CURE can also edit nuclear RNAs.

Recently, Merkle *et al*. developed a guide RNA (gRNA) that recruits endogenous human ADAR2 to induce programmable site-specific A-to-I editing in RNA (Merkle *et al.* 2019). These gRNAs consist of two parts: a conserved ADAR recruitment domain and a programmable specificity domain. This system is referred to as Recruiting Endogenous ADAR to Specific Transcripts for Oligonucleotide-Mediated RNA Editing (RESTORE) (Merkle *et al.* 2019). Qu *et al*. independently developed short-engineered ADAR recruitment RNAs (arRNAs) to recruit endogenous ADAR1 or ADAR2 enzymes, thereby converting specific adenosines to inosines in RNA. This approach is named Leveraging Endogenous ADAR for Programmable Editing of RNA (LEAPER) (Qu *et al.* 2019). LEAPER exhibits high editing specificity with rare off-target mutations and limited bystander editing. Reautschnig *et al*. optimized the design of gRNAs by combining the target site of the gRNA with a cluster of recruiting sequences (RS) freely distributed across the target RNA and named these gRNAs CLUSTER gRNAs (Reautschnig *et al.* 2022). This CLUSTER design resulted in gRNAs with high sequence flexibility and enabled efficient RNA A-to-I editing both in cultured cells and *in vivo* with significantly reduced bystander editing. Recently, Katrekar *et al*. and Yi *et al*. employed covalently closed circular arRNAs, named cadRNAs and LEAPER 2.0, to further enhance editing efficiency and reduce bystander editing (Katrekar *et al.* 2022; Yi *et al.* 2022). Though DNA editing in the genome can potentially provide long-lasting and even permanent cures, it comes with the potential risks of long-lasting off-target effects. In contrast, RNA editing offers tunability and reversibility as it does not cause permanent changes in the genome. Therefore, RNA editing has unique advantages in certain therapeutic contexts.

## PERSPECTIVE

Since the first series of BEs were developed in 2016, BEs have become revolutionary gene editing tools (Gaudelli *et al.* 2017; Komor *et al.* 2016). Great efforts have been made to develop modular BEs with high precision and efficiency (Huang *et al.* 2021a; Kim and Chen 2023; Yang *et al.* 2019; Yang and Chen 2020). In this review, we have described different types of nucleoside deaminases that are the key effectors of these revolutionary gene editing tools, with an emphasis on their structures and functionality. The high-resolution structure of the base editor machine detailed the interfaces between deaminase and their nucleotide substrates, which provide the blueprint for subsequent rational design and engineering of deaminases to improve the efficiency of corresponding base editors and reduce off-target risks. Besides structure-guiding rational design, directed evolution of deaminase is also a classic route and has achieved great success, such as TadA-8e and ADAR2 in RESCUE, which shall remain an important path for deaminase optimization in the future. More recently, AI-assisted structural classification has also been successfully applied to discover novel ssDNA and dsDNA cytidine deaminases, suggesting that AI-based discovery of new tool enzymes is also a novel and effective method, besides directed evolution and structure-guided rational engineering of known proteins.

Though current BEs have realized targeted editing of nucleic acid substrates in various contexts, new BEs are still needed to achieve specific and unlimited base editing at all desired sites. Such new BEs can be generated by discovering new nucleoside deaminase families and engineering the vast pool of nucleoside deaminases. We envision that these new BEs will be broadly used in biotechnology, basic research, and translational medicine in the future.

## Conflict of interest

Jiangchao Xiang, Wenchao Xu, Jing Wu, Yaxin Luo, Bei Yang and Jia Chen declare that they have no conflict of interest.
